# Circular Array Fiber-Optic Sub-Sensor for Large-Area Bubble Observation, Part I: Design and Experimental Validation of the Sensitive Unit of Array Elements

**DOI:** 10.3390/s25206378

**Published:** 2025-10-16

**Authors:** Feng Liu, Lei Yang, Hao Li, Zhentao Chen

**Affiliations:** Meteorological Oceanographic College, National University of Defense Technology, Changsha 410073, China; ylei0731025@126.com (L.Y.); lihao_pla@163.com (H.L.); czt1212@126.com (Z.C.)

**Keywords:** bubble, buoy-type bubble sensor, a circular array fiber-optic sub-sensor, sensitive unit of array elements

## Abstract

For large-scale measurement of microbubble parameters on the ocean surface beneath breaking waves, a buoy-type bubble sensor (BBS) is proposed. This sensor integrates a panoramic bubble imaging sub-sensor with a circular array fiber-optic sub-sensor. The sensitive unit of the latter sub-sensor is designed via theoretical modeling and experimental validation. Theoretical calculations indicate that the optimal cone angle for a quartz fiber-optic-based sensitive unit ranges from 45.2° to 92°. A prototype array element with a cone angle of 90° was fabricated and used as the core component for feasibility experiments in static and dynamic two-phase (gas and liquid) identification. During static identification, the reflected optical power differs by an order of magnitude between the two phases. For dynamic sensing of multiple microbubble positions, the reflected optical power varies from 13.4 nW to 29.3 nW, which is within the operating range of the array element’s photodetector. In theory, assembling conical quartz fiber-based sensitive units into fiber-optic probes and configuring them as arrays could overcome the resolution limitations of the panoramic bubble imaging sub-sensor. Further discussion of this approach will be presented in a subsequent paper.

## 1. Introduction

Bubble plumes generated by breaking waves (hereafter “bubble plumes”) play a significant role in various oceanic and atmospheric processes. They enhance air–sea gas exchange [1,2,3], reduce sound propagation speed in the upper ocean [4], and accelerate acoustic scattering and attenuation [5]. Additionally, they influence sonar transmission [6] and ocean optical remote sensing [7]. However, precisely quantifying their contributions through in situ measurements remains a major challenge due to their spatiotemporal complexity. Bubble plumes evolve rapidly, span the sea surface and subsurface, and contain a broad range of bubble sizes. Their surface coverage typically extends only a few square meters, yet subsurface penetration can reach 10 m or more [8,9]. Wave breaking produces densely packed large bubbles within microseconds, which quickly fragment into clouds of microbubbles that may persist in the upper ocean for several seconds. Bubble diameters during this process can range from tens of micrometers to several centimeters [10,11,12]. Accurate in situ measurements of bubble plumes, therefore, require instruments capable of high-frequency response and fine bubble size resolution, while also covering a wide vertical domain across the air–sea interface.

To meet stringent temporal and spatial resolution requirements, current offshore observational campaigns often employ arrays of identical bubble sensors for multi-point measurements of bubble plume parameters. Alternatively, instruments based on different measurement principles, such as imaging, acoustics, and fiber optics, are combined to assess the composite characteristics of bubble plumes across multiple locations. For example, during a North Pacific expedition in December 2019, Morteza Derakhti et al. [13] deployed an array of 2–6 SWIFT buoys [14] for synchronous measurements of breaking gravity waves and associated bubble plumes. Each buoy was equipped with a Doppler sonar to capture acoustic backscatter data from the plumes. Through signal processing, including thresholding and filtering, the team quantified the penetration depth and residence time of the bubble plumes. Similarly, during Cruise D320 of the UK-SOLAS program [15], researchers used acoustic and fiber-optic instruments to monitor bubble cloud injection during wave breaking. An 11-m mast-mounted buoy carried acoustic transducers and hydrophones spanning 3–200 kHz, detecting bubbles ranging from approximately 16 mm to 1141 mm in diameter. A fiber-optic probe simultaneously recorded bubble plumes at an average depth of 0.25 m. In the North Atlantic High Wind Gas Exchange Study (HiWinGS) near Greenland [11,12], three types of instruments, a bubble-specific camera, an acoustic resonator, and an upward-looking sonar, were mounted on the same buoy. The sonar, deployed at 8 m, monitored temporal variations in bubbles via acoustic backscatter, tracking plume evolution. The resonator at 4 m and the bubble camera at 2 m simultaneously measured void fraction and bubble size distribution at different depths. Results confirmed that all three instruments successfully detected bubble plumes, while also highlighting the limitations of relying on a single instrument. For instance, although the sonar detected strong backscatter signals corresponding to high void fractions at 2–4 m, the bubble camera and resonator did not capture these high-void plumes.

Previous studies have primarily relied on buoys equipped with multiple instruments deployed at different depths, representing a typical vertical multi-point measurement approach. This method is highly effective for capturing underwater bubble plume characteristics during early-stage development, where parameters such as entrainment depth and residence time dominate physical processes, including air–sea gas exchange. These parameters exhibit significant variations with respect to depth and are highly sensitive to instrument response, making them primary targets during instrument design. However, the effectiveness of this approach declines when observing microbubble populations generated at the sea surface during later plume stages. Surface microbubbles cover large areas and occur in high abundance, but vertical multi-point methods are limited to single-point monitoring, restricting coverage and sampling efficiency. For example, in the UK-SOLAS program [15,16,17], three representative North Atlantic cruises scheduled five observation stations each. Deploying a bubble video measurement system required 1–4 h per station, sampling a volume of 20 × 2.9 × 1.9 mm^3^. Images were collected every 5 min over 2-min acquisition periods, resulting in single-point sampling durations of 1–2 h. Extreme weather further limited data collection: during the D313 cruise, the system was successfully deployed once, yielding just 154 image datasets across all three cruises.

**Figure 1:**
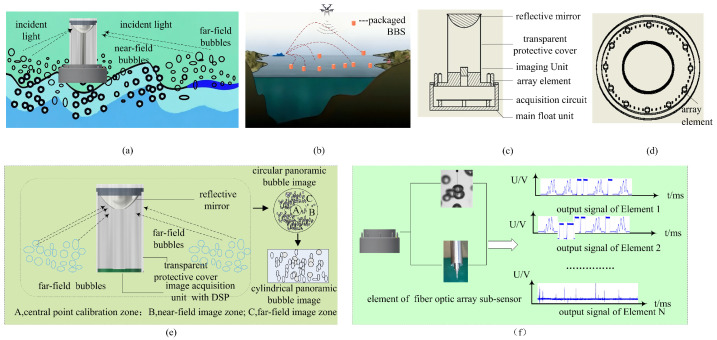
Schematic diagram of BBS measurement principle. (**a**) Schematic diagram of BBS measurement principle; (**b**) expendable bubble plume observation array based on BBS; (**c**) cross-sectional view of BBS; (**d**) top view of BBS; (**e**) schematic diagram of the panoramic bubble imaging sub-sensor; (**f**) schematic diagram of the circular array fiber-optic sub-sensor.

To address this issue, a novel buoy-type in situ bubble observation sensor (BBS) was designed to expand the single-point coverage of existing bubble measurement instruments. The BBS allows the deployment of an array at the sea surface for multi-point monitoring of surface bubble populations that form during the later stages of plume evolution. This sensor is typically applied in an expendable bubble plume observation array based on BBS, as shown in Figure 1b. BBS comprises a panoramic bubble imaging sub-sensor and a circular array fiber-optic sub-sensor. The panoramic imaging system captures bubble field information over a long visual range, while the circular fiber-optic array monitors the near-field region, compensating for detection “blind spots” in dense bubble clusters that the panoramic system cannot cover. This paper focuses on the design optimization and experimental validation of the sensitive units within the circular array fiber-optic sub-sensor. Section 2 describes the working principle of the BBS; Section 3 details the detection principle and theoretical design basis of the circular array’s sensing elements; and Section 4 presents prototyping, experimental validation, and results analysis. Once integrated with data acquisition circuitry, these sensing elements are encapsulated into fiber-optic probes, forming the array elements that enable multi-point measurement of dense near-field bubble clusters. The detailed development and deployment of this component will be addressed in a subsequent paper.

## 2. BBS Measurement Principle

The measurement principle of the BBS system is illustrated in Figure 1. It comprises a panoramic bubble imaging sub-sensor, a circular array fiber-optic sub-sensor, and the BBS packaging structure. The panoramic bubble imaging sub-sensor is built on a refractive–reflective panoramic imaging system. As shown in Figure 1e, it comprises two core components: a high-resolution image acquisition unit centered on a DSP controller and a coaxially aligned reflector. The reflector directs a 360° image of the BBS surroundings toward the image sensor, producing a circular omnidirectional bubble image. This image is then reconstructed into a cylindrical panoramic bubble image using a dedicated processing algorithm. The reflector enables coverage of a 360° horizontal and over 180° vertical field of view, capturing a broad expanse of the water column. However, it introduces significant image distortion in the central, near-field region (Figure 1a). To address this limitation, the circular array fiber-optic sub-sensor performs “compensatory acquisition” in the near-field. As illustrated in Figure 1c,d, it consists of multiple fiber-optic probe elements, with the number of elements tailored to the detection area. Each probe generates a distinct bubble pulse signal sequence upon interaction with bubbles, owing to the differing reflectance characteristics of liquid and gas phases (Figure 1f). When deployed on a drifting buoy equipped with a high-speed image transmission interface that also supports high-volume, precisely synchronized pulse signal transmission, the two sub-sensors operate in tandem. The panoramic imaging sub-sensor captures far-field cylindrical images, while the fiber-optic sub-sensor records near-field pulse sequences. From the panoramic images, parameters such as water surface area, bubble count, and bubble size distribution in the far field can be derived, while the pulse sequences provide corresponding near-field information. Together, these measurements enable large-area, high-resolution observation of surface-layer bubble populations from a single sampling event, fulfilling the requirements for marine surface bubble monitoring applications.

## 3. Optimization Method for the Sensitive Units of Circular Array Fiber-Optic Sub-Sensor Elements

### 3.1. Subsection Measurement Principle of Array Elements

**Figure 2:**
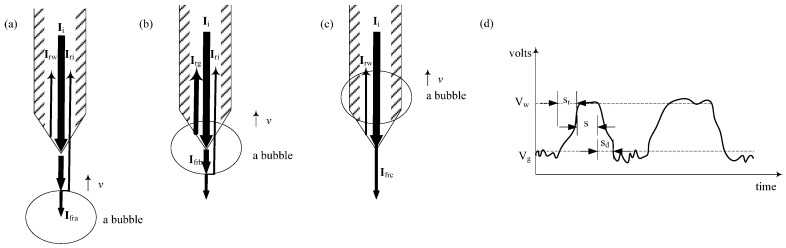
Measurement principle of circular array fiber-optic sub-sensor elements. (**a**) Approaching the bubble field; (**b**) in contact with the bubble field; (**c**) departing the bubble field; (**d**) typical signal of circular array fiber-optic sub-sensor elements.

The optical system of the panoramic bubble imaging sub-sensor mainly comprises a reflective mirror, an imaging unit, and a transparent protective cover (Figure 1b). The mirror reflects the bubble objects surrounding the BBS, forming images on the detector plane of the imaging unit. The transparent protective cover provides structural support and protection for the mirror and imaging unit. The main float serves primarily to house the data acquisition circuitry in a watertight compartment and acts as a mounting platform for types of sub-sensors. It can be dynamically connected to drifting or other types of buoys. The elements of the circular fiber sub-sensor array are uniformly distributed around the main float at 30° intervals in the horizontal azimuth (Figure 1c). Each array element adopts a fiber probe as its structural prototype. The measurement principle of each array element is depicted in Figure 2. Assuming that the direction of bubble motion is parallel to the longitudinal axis of the fiber probe, the fiber probe detects the bubble in three typical scenarios depending on the spatial relationship between the bubble and the probe: (a) approaching the bubble field, (b) in contact with the bubble field, and (c) departing the bubble field. In all three scenarios, an incident light beam with intensity Ii is emitted by the light source. The beam undergoes varying degrees of reflection and refraction at the fiber probe tip, the upper boundary of the bubble, and the lower boundary of the bubble. Ignoring incident light absorption in gas and liquid phases and reflected light attenuation during transmission, the reflected light intensities returning to the fiber probe are as follows:

(1) $$I_{ra}=I_{rw}+I_{ri}=I_{i}-I_{fra}$$

(2) $$I_{rb}={\;I}_{rg}+I_{ri}=I_{i}-I_{frb}$$

(3) $$I_{rc}=I_{rw}={\;I}_{i}-I_{frc}$$

where $I_{\mathrm{ra}}$, $I_{rb}$, and $I_{rc}$ denote the reflected light intensities under the three respective scenarios. $I_{rw}$ and $I_{rg}$ represent the reflected light intensities generated at the probe end face when incident light rays enter the liquid phase and gas phase. $I_{ri}$ is the reflected light intensity corresponding to the incident light rays at the upper and lower boundaries of the bubble. $I_{fra}$, $I_{frb}$, and $I_{frc}$ denote the refracted light intensity corresponding to three typical scenarios. In practical detection environments, the incident beam is not perfectly parallel to the direction of bubble motion. Moreover, bubbles exhibit irregular shapes, deviating from ideal spheres or ellipsoids, resulting in deformation at the upper and lower boundaries. As a result, incident light may reflect in various directions at the bubble boundary. The reflected light intensity, $I_{ri},$ is significantly lower than the reflection intensities $I_{ra}$, $I_{rb}$, and $I_{rc}$ and can therefore be neglected. Therefore, the ratio of reflected light intensity to incident light intensity in Equations (1)–(3) can be expressed as

(4) $$Ra\;={\;R}_{c}\;\text{≠}\;R_{b}$$

By neglecting the difference between the incident angle within the fiber core and the refracted angle in the external medium for the local normal at the fiber tip interface, Equation (4) can be rewritten in terms of the refractive indices of the three scenarios:

(5) $$R_{a}\;={\;R}_{c}\;={\;\frac{\left(n_{fi}-n_{w}\right)}{{\left(n_{fi}+n_{w}\right)}^{2}}}^{2}$$

(6) $$R_{b}={\;\frac{\left(n_{fi}-n_{g}\right)}{{\left(n_{fi}+n_{g}\right)}^{2}}}^{2}$$

where $n_{fi}$, $n_{w},$ and $n_{g}$ represent the refractive indices of the fiber, water, and air. The fiber probes used in this study are made of single-mode quartz fiber, with a refractive index of 1.44. The refractive indices of water and air are 1.33 and 1.00. Substituting these values into Equations (5) and (6) allows for the calculation of the reflection coefficients corresponding to the three typical detection scenarios: $R_{a}\;=\;R_{c}\;=\;0.00158$ and $R_{b}\;=\;0.0325$.

As a result of the differing reflection coefficients in the three scenarios, when a photodetector is installed at the end of the fiber probe, it detects varying optical powers. These powers are converted into electrical signals with different peak current/voltage values via photoelectric conversion (Figure 2d). By selecting the appropriate threshold values V_g_ and V_w_, it is possible to distinguish between liquid and gas phases. When the bubble signal frequency varies, the photodetector outputs a pulse sequence comprising a rising-edge signal S_r_ corresponding to the gas-to-liquid transition, a stable signal S corresponding to the liquid phase, and a falling-edge signal S_d_ corresponding to the liquid-to-gas transition. By analyzing the rising edge timing and calibrating bubble velocity, calculating the duration of the gas phase, determining the falling edge timing and calibrating again for velocity, and computing pulse frequency, one can extract bubble velocity, bubble diameter, and bubble count. The calculation of bubble plume parameters from the back-reflected optical power signal obtained in this study primarily involves the following steps: (1) converting back-reflected optical power signal into a voltage signal and (2) transforming the voltage signal into bubble plume parameters. The first step can be accomplished through hardware components (photoelectric converter, analog-to-digital converter, and controller). The second step is categorized based on the number and arrangement of array elements, including three typical processing methods: single-array-element bubble signal processing, dual-array-element bubble signal processing, and quad-array-element bubble signal processing. The outlines of these three typical algorithms are provided in the Appendix A.

### 3.2. Key Parameter Calculation of the Sensitive Unit in the Array Element

**Figure 3:**
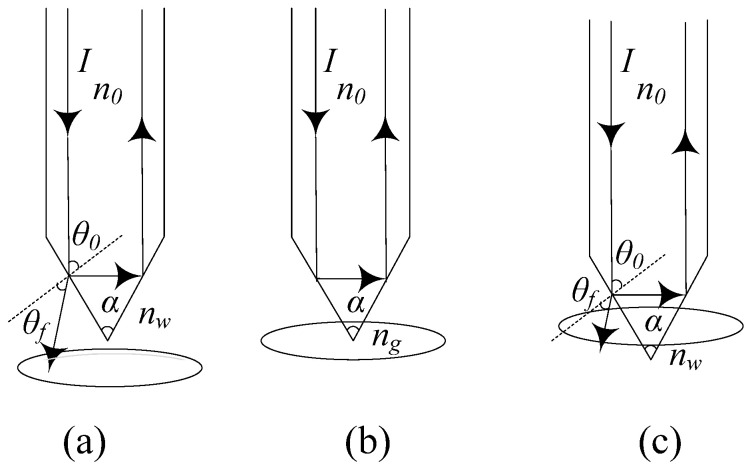
Interaction mechanism between the array element and the bubble: (**a**) approaching the bubble field; (**b**) in contact with the bubble field; (**c**) departing the bubble field.

The sensing performance of the fiber-optic end face at the front end of the array element directly affects the overall detection performance of the array element (Figure 2a–c). Fabricating the fiber end face into a conical fiber lens is most advantageous for piercing gas bubbles and facilitating coupling with the transmission of the rear-end fiber optic [18,19,20]. Therefore, we adopt a conical fiber lens as the structural prototype for the sensitive unit at the front end of the array element. This study focuses on analyzing how variations in the cone angle affect optical power, aiming to determine the optimal processing range for the array element. When the conical lens at the fiber end face of the array element comes into contact with the gas and liquid phases of a bubble, the incident light ray I with an angle of incidence $\theta_{0}$ undergoes refraction at the conical lens (Figure 3). A portion of the light is refracted into the water at a refraction angle $\theta_{f}$, while another portion is reflected into the array element. According to Equations (5) and (6), the greater the difference in returned optical power between the liquid and gas phases at the rear detector of the array element, the easier and faster the phases can be distinguished. As a bubble rises along the main axis of the array element, the contact area between the conical lens and the gas phase increases. According to the principles of reflection and refraction, more incident rays undergo total internal reflection within the bubble, directing the light entirely into the array element and causing the detector to register the maximum returned optical power. The corresponding optical power difference also reaches its peak. The interaction mechanism between the array element and the bubble can be described by Equation (7) (Figure 3):

(7) $$sin\theta_{0}n_{fi}=sin\theta_{f}n_{w}$$

where $\theta_{0}$ denotes the incident angle of the incoming light, $n_{fi}$ is the refractive index of the conical fiber lens, $\theta_{f}\;$ is the refraction angle, and $n_{w}$ refers to the refractive index of water. When total internal reflection occurs in the gas phase, Equation (7) becomes

(8) $$sin\theta_{0}n_{fi}=n_{w}$$

According to the relationship between the incident angle and the cone angle of the fiber-optic probe,

(9) $$\alpha =2\left(90\text{°}-\theta_{0}\right)$$

After substituting Equation (9) into Equation (8) to rapidly distinguish between the liquid and gas phases, the cone angle of the conical lens “α” must satisfy the following condition:

(10) $$2{cos}^{-1}\frac{n_{g}}{n_{fi}}\;<\;\alpha \;<\;2{cos}^{-1}\frac{n_{w}}{n_{fi}}$$

Referring to the material of the fiber-optic probes (sapphire fiber) currently used in open-sea bubble plume observation experiments and comparing it with the commonly used quartz fiber in fiber-optic sensors, quartz fiber offers the typical advantages of a well-established fabrication process and lower cost. These characteristics make it particularly suitable for the typical observation application scenario discussed in this study—an expendable observation array based on BBS. Hence, the material selected for the tapered-lens fiber-optic probe is quartz fiber. By substituting the refractive indices of quartz fiber, air, and water into Equation (10), the theoretical value of the cone angle for the conical lens is determined to satisfy the condition: 45.2° < α < 92°.

## 4. Fabrication and Performance Testing of Array Elements

### 4.1. Fabrication of Array Elements

**Figure 4:**
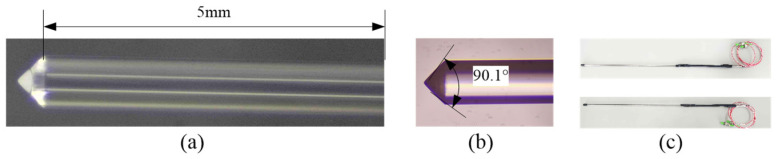
Array elements: (**a**) the length of the bare fiber; (**b**) the cone angle of the array element; (**c**) two array elements integrated with a fiber coupler.

Our study leveraged a single-mode quartz fiber optic featuring a core diameter of 8 μm and a cladding diameter of 125 μm. A tapered-lens fiber probe was fabricated using the fiber-polishing technique in four stages: pre-treatment, pre-assembly, polishing, and encapsulation. The dimensions of the fabricated probes are shown in Figure 4. Using a 50 cm long probe as an example, the pre-treatment stage involved stripping a 5 cm section of the fiber’s protective coating with a fiber stripper, followed by cleaning with alcohol. During pre-assembly, stainless steel needles of 1 mm, 3.1 mm, and 5.3 mm diameters were cut, polished, and sequentially pre-assembled using heat-shrink tubing. A 5 mm section of the 1 mm diameter bare fiber remained exposed to allow proper insertion into the ceramic ferrule for polishing. The heat-shrink tubing was secured by heating the ends. Polishing proceeded in three stages—coarse grinding, fine grinding, and final polishing—achieved by adjusting the rotation speed and grinding time of a fiber-polishing machine and using abrasive films of decreasing grit sizes. For coarse grinding, 3 μm abrasive paper was attached to an alcohol-cleaned polishing disk, and the ferrule-mounted fiber was polished at an appropriate angle under light pressure for approximately one minute. For fine grinding, we employed a 1 μm diamond polishing film at a higher rotation speed for about one minute. Final polishing was finished using 0.1 μm abrasive paper at an increased speed for 15 s. The physical appearance and detailed images of the fabricated array elements are presented in Figure 4. To facilitate performance testing, the fiber coupler was integrated with the fiber probe. The integrated fiber probe is shown in Figure 4c. Two array elements were successfully fabricated.

### 4.2. Experimental System Setup

**Figure 5:**
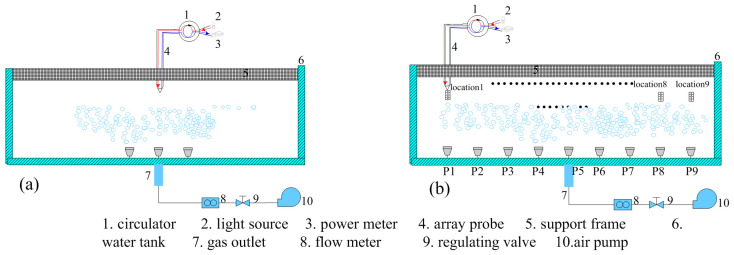
Schematic diagram of the experimental setup. (**a**) Static phase recognition test; (**b**) dynamic phase recognition test.

To verify the feasibility of the cone-shaped-lens fiber-array structure based on quartz fiber optics for detecting high-density bubble plumes, an experimental system was established based on measurement principles and fabricated array elements. The system included a light source, fiber-optic circulator, optical power meter, array element support frame, and a bubble plume simulation device. We evaluate the static identification of gas and liquid phases and the dynamic recognition of bubble plumes. The bubble plume simulation device comprised a water tank, air pump, flow meter, adjustable air nozzles, and a flow control valve (Figure 5). The water tank (0.6 m × 0.5 m × 0.5 m) was made of 3 mm thick PMMA with a visible to near-infrared light transmittance of 92%. By properly adjusting the flow meter and control valve and selecting air nozzles of various sizes, bubble diameters in the range of 0.15 mm to 0.5 mm could be generated. The light source was a 1310 nm pigtail-type laser (model ZH-S13D1EA10Y1), with a maximum output power of 10.36 mW and a maximum operating current of 47 mA. The fiber-optic circulator operated within a wavelength range of 1310 ± 30 nm, with maximum insertion losses of 0.83 dB and 0.77 dB between the three ports. The corresponding isolation values are 0.83 dB and 0.77 dB. The optical power meter (model JC3118) featured a sampling rate of 1 kHz and a wavelength range of 800 nm to 1700 nm. It supported data output to a computer and was equipped with an InGaAs detector. To enhance the coupling efficiency between the circulator and the fiber-optic probe, an integrated packaging was employed (the black section in Figure 4c). The optical path of the light source is indicated by red and blue arrows (Figure 5). Light emitted from the source was transmitted via the fiber coupler to the conical lens end of the fiber probe. The reflected light traveled back through the fiber to the coupler and was transmitted to the optical power meter for detection of the reflected light intensity.

### 4.3. Experimental Results

**Figure 6:**
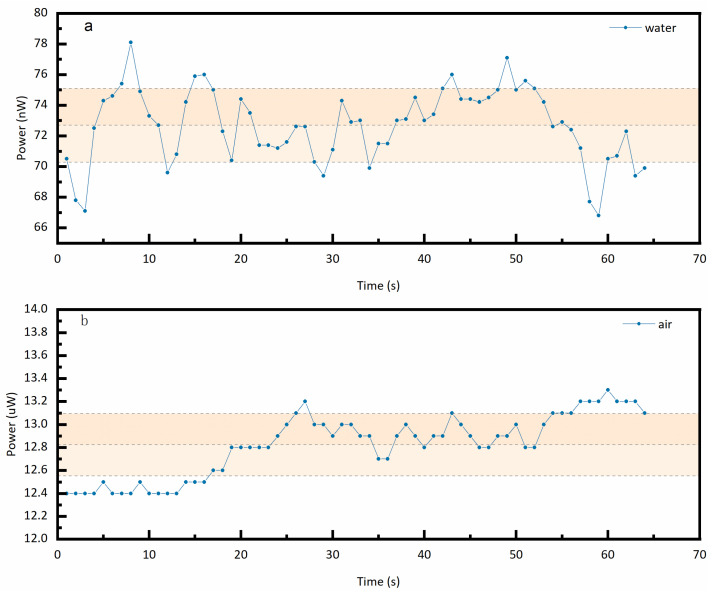
Back-reflected optical power during static phase recognition (sampling duration: 64 s). (**a**) in water; (**b**) in gas.

**Figure 7:**
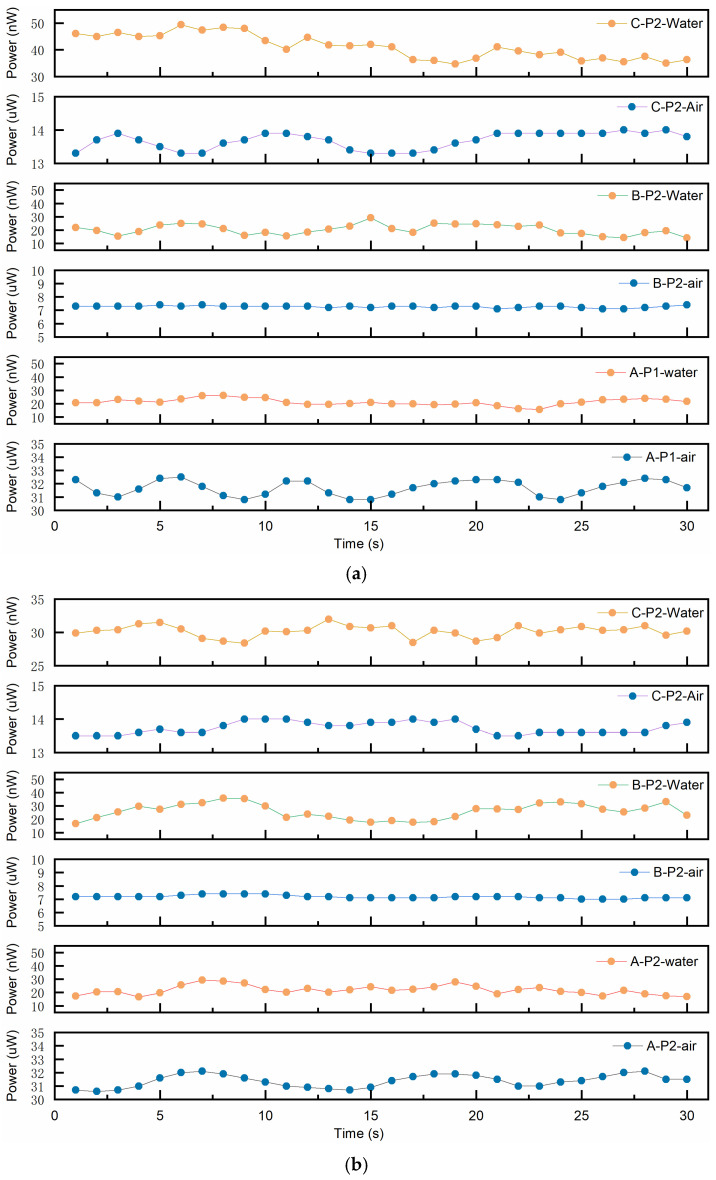
Back-reflected optical power during static phase recognition (three repeated trials with 30 s sampling duration): (**a**) P1 probe; (**b**) P2 probe.

**Figure 8:**
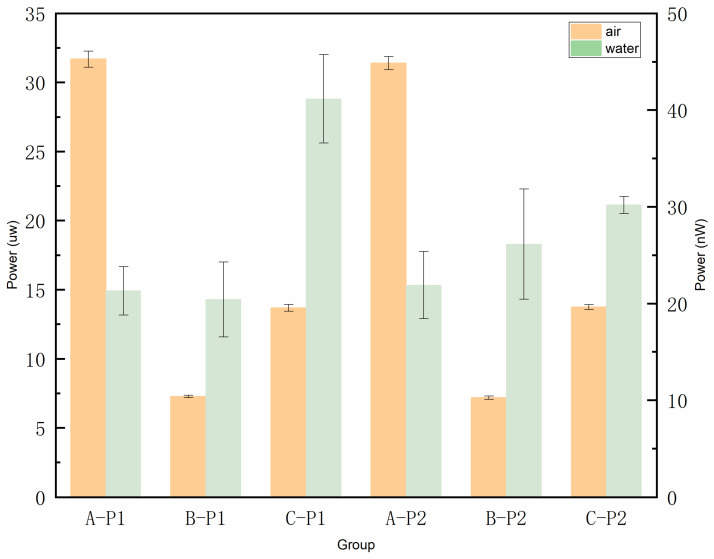
Back-reflected optical power during static phase recognition (three repeated trials with 30 s sampling duration were presented as mean ± standard deviation).

First, static phase identification tests for the gas phase and liquid phase were conducted on the fabricated fiber-optic probes. The two trial-fabricated fiber-optic probes were placed in air and water, respectively, with sampling and interval durations of no less than 30 s in each medium. The optical power meter was set to a sampling frequency of 1 kHz, and data were transmitted to the computer via its communication interface every 100 ms. According to the above settings, the static test procedure consisted of first performing continuous sampling in the liquid and gas phases for no less than 1 min, followed by three sets of repeated 30-s interval sampling. During the 1-min continuous sampling, both probes operated simultaneously. For the 30-s-interval experiments, each group of measurements was separated by an interval of 1 min and 30 s. Figure 6 shows the average return light power distribution of the two probes in both liquid phase and gas phase with sampling duration of 64 s. This figure presents the data of 64 return light power after averaging every 1 s. The return light power distribution of the two fiber probes in the gas phase ranges from 12.4 μW to 13.3 μW, with an average of 12.82 μW. The reflected power distribution in the liquid phase ranges from 66.8 nW to 78.1 nW, with an average of 72.69 nW. Figure 7 shows the reflected light power data of probe 1 and probe 2 continuously sampled for 30 s in the liquid phase and gas phase, respectively, including three sets of repeated experiments. Under the initial 30 s (i.e., Group A), the reflected light power distribution detected when the probe was in the gas phase was 30.8 μW to 32.5 μW and 30.6 μW to 32.1 μW, and when located in the liquid phase, the reflected light power distribution is between 15.7 nW and 26.1 nW and 16.7 nW and 29.3 nW. The output errors of the two array elements in the same environment are relatively small, preliminarily indicating a good consistency between the two array elements. The difference in the reflected light power of the two probes in the liquid phase and the gas phase shown in Figure 8 reaches one order of magnitude, which initially indicates that the trial-fabricated array element can distinguish between the static gas phase and the liquid phase. Moreover, in this figure, the results of the repeated experiments are presented as mean ± standard deviation. The fluctuation trends of the reflected light power obtained from the three groups of experiments conducted by the two probes are similar. For instance, the reflected light power in the gas phase of probe 1 under the initial 30 s (i.e., Group A) varies according to (31.6 ± 0.58) uW, (7.27 ± 0.08) uW, and (13.68 ± 0.25) uW. Under the same conditions, the reflected power corresponding to probe 2 changed according to (31.3 ± 0.47) uW, (7.10 ± 0.12) uW and (13.70 ± 0.18) uW. The two probes also showed a similar changing trend in the subsequent two 30 s (i.e., Group B and Group C), further indicating that the consistency of the two array elements was good. Although the two array elements demonstrated good consistency—attributed to simultaneous probe polishing, the use of fiber couplers from the same batch, and testing under identical conditions—their repeatability was unsatisfactory. For instance, the three gas-phase reflection power measurements for probe 1 were (31.6 ± 0.58) μW, (7.27 ± 0.08) μW, and (13.68 ± 0.25) μW, showing considerable fluctuation. This variability is mainly due to the short intervals between experiments, which prevented complete dehumidification of the probes after liquid-phase testing.

Next, bubble plumes were detected using the fiber-optic probe via dynamic phase recognition experiments. An air pump (Figure 5) generated gas flow through nine needle-shaped outlets with different diameters to simulate microbubble groups. The outlet diameters were 0.15 mm (P1), 0.17 mm (P2), 0.2 mm (P3), 0.22 mm (P4), 0.25 mm (P5), 0.27 mm (P6), 0.3 mm (P7), 0.4 mm (P8), and 0.5 mm (P9), with 3 cm spacing between outlets. The water tank depth was 20 cm. When all nine outlets were activated, a microbubble detection zone formed on the water surface. The fiber-optic probe, vertically mounted using the support frame (Figure 5), measured back-reflected power at nine points (location 1 to location 9) (±1 cm from bubble outlets). Figure 8 presents the real-time back-reflected power data collected over one minute at the nine positions. Compared to the static phase tests, the dynamic recognition of bubble plumes showed back-reflected power variations in the nanowatt range for phases, with a smaller difference between the gas and liquid phases. This is mainly due to the much shorter dehumidification time in the presence of dynamically changing bubbles. During the transition, a mixed-phase state often occurs. 

**Figure 9:**
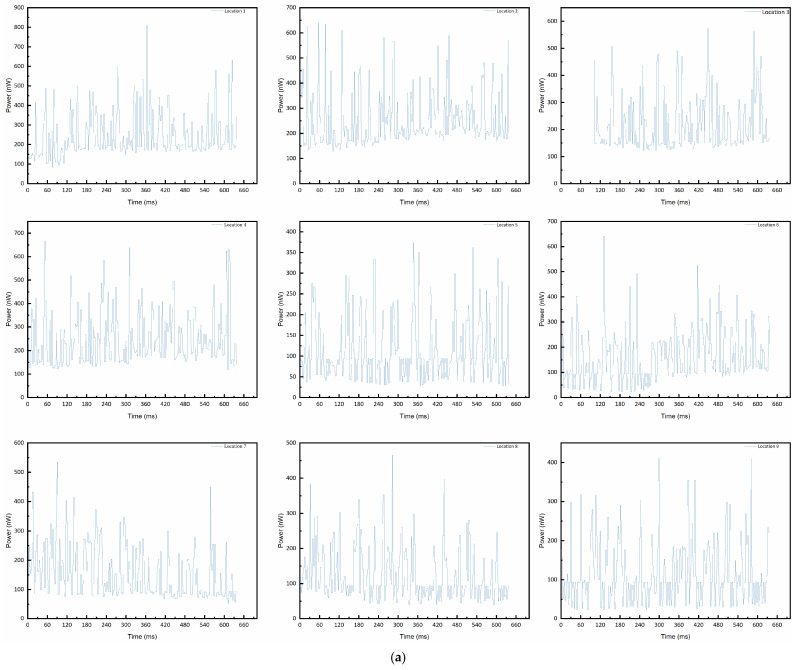

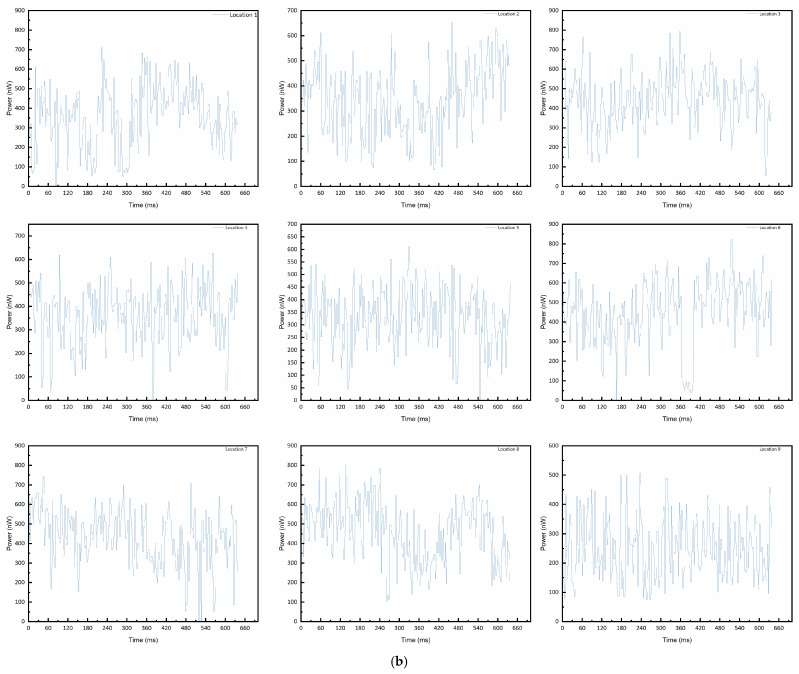
Dynamic test signals of the probe at different measurement positions: (**a**) A group; (**b**) B group.

**Figure 10:**
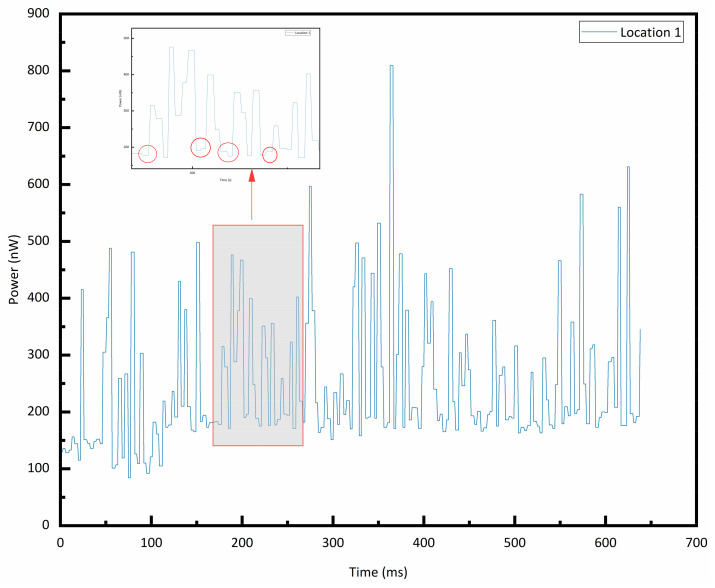
Local magnification of the test signals corresponding to the probe.

The results of Figure 9 robustly confirm that our measured signals, while small, represent a true physical response. Figure 10 (a magnified segment from position 1) shows these transitions, with red circles indicating their varying conversion durations. After calculating the signal-to-noise ratio (SNR) of each signal, it was confirmed that the signals are indeed relatively weak. The primary reasons include the following:

- The bubble size is excessively small or the interfacial curvature is excessively large. The signal strength measured by the probe is closely related to the interfacial curvature between the bubble and the liquid. Excessively small bubbles or sharp curvature may lead to suboptimal reflection/scattering patterns of light, resulting in minimal variation in the light intensity entering the receiving optical fiber (i.e., a very weak signal).
- The probe failed to penetrate the bubble in an ideal manner (e.g., perpendicularly), instead skimming over or partially penetrating it. This results in less distinct optical changes at the interface and a reduction in signal amplitude.
- Ambient light interference: Ambient light in the experimental environment (especially indoor lighting) can be detected by the probe, creating a strong and stable DC offset background. Fluctuations in this background (e.g., due to inherent instability of the light source or reflections caused by liquid surface disturbances) directly superimpose onto the measurement signal, resulting in significant noise.

**Figure 11:**
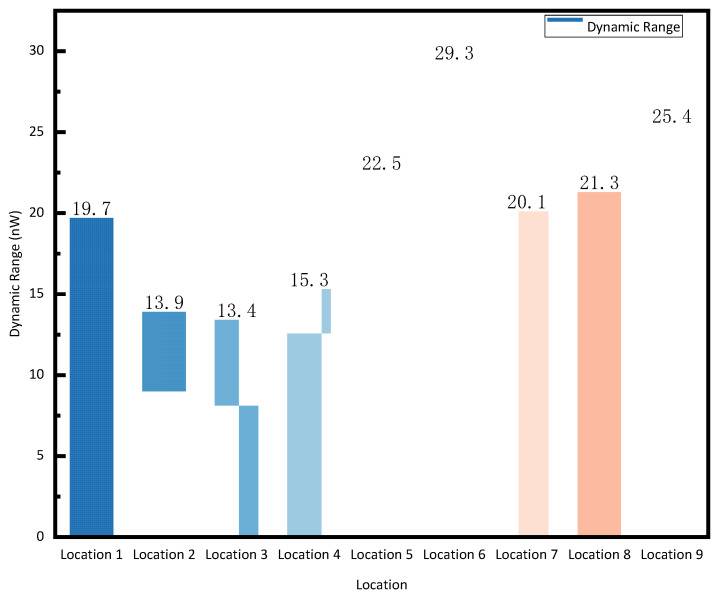
Dynamic range of reflected optical power at nine typical measurement points.

Figure 11 presents a statistical analysis of the dynamic range of reflected optical power at nine typical measurement points for the probe. The dynamic range spans from 13.4 nW to 29.3 nW. This range is suitable for converting the optical signals returned by the probe elements into electrical signals using optoelectronic conversion devices: avalanche photodiodes (APDs) and photomultiplier tubes (PMTs). Furthermore, this dynamic range can be used to determine the appropriate range settings for the aforementioned photodetectors. The dynamic range of reflected power between adjacent measurement positions shows the following characteristics: the maximum dynamic range is observed near the central region, followed by the lateral positions. In particular, the differences in dynamic range at central positions (location 4 to location 6) reach up to 6.8 nW and 7.2 nW, while those at lateral positions (location 1 vs. location 2, and location 8 vs. location 9) are 5.8 nW and 4.1 nW. This disparity is likely due to the merging of bubble plumes in the central area causing greater variation. In contrast, bubbles at the sides are closer to the tank walls, and their interactions or collisions with the boundaries tend to introduce greater variability in the measurements.

## 5. Discussion

This study first explores the use of standard quartz fiber optics processed into tapered lenses as the sensing unit of fiber-optic probes. It aims to construct a probe array coupled with an image sensor for jointly measuring the size distribution of microbubble clusters near the ocean surface. The focus is on the feasibility of using the probe’s sensing unit to detect bubble parameters, which constitutes part of the sensor unit design and validation for a BBS system. Therefore, comparing the findings with previous studies on fiber-optic probes for measuring oceanic bubble plumes is difficult. Nevertheless, from the perspective of feasibility and the material composition of the sensing units, it remains meaningful to compare this study with the laboratory and offshore observations conducted by the teams of Serdula [19] and Blenkinsopp [20,21], which utilized sapphire fiber probes. They targeted the early evolution of high-density bubble plumes generated during the initial air entrainment process, featuring large bubble sizes and rapid dynamic changes. Therefore, they employed sapphire fiber probes with a high refractive index of 1.76. The observed durability of the sapphire probe, coupled with its well-known material properties, suggests it may offer a superior dynamic response performance over quartz-based probes in scenarios involving capturing the fast-evolving characteristics of high-density bubble plumes. In contrast, this study targets slower, long-lived microbubble clusters, for which the chosen quartz fiber is well-suited. This is validated by a reflected power difference of an order of magnitude between phases. While other teams use 1 mm sapphire probes to minimize disturbance in large-bubble plumes (e.g., up to 64 mm), this study’s 125 μm probe limits interference in millimeter-scale flows. Nevertheless, for detecting microbubbles in the 0.1 mm to 1 mm range, improving the resolution of the optoelectronic conversion process and phase discrimination methods between liquid and gas phases is essential to ensure consistent resolution from 0.1 mm to several millimeters. For instance, techniques like the absolute amplitude threshold method (using either single or dual thresholds) proposed by Barrau et al. or adaptive thresholding (i.e., dynamically adjusting thresholds according to signal amplitude and shape [22] may be adopted). More advanced waveform analysis could also be employed to extract the amplitude and shape characteristics of each bubble signal. Using a two-point discrimination method to monitor local extrema could enable reliable differentiation between gas and liquid phases for each bubble signal.

Although the sensitive unit of the array element prototype proposed in this paper can achieve preliminary expected design objectives, the completed tests still need to be conducted after system integration. The preliminary test results obtained after system integration are shown in Appendix B. According to the initial test results, integrated BBS still faces many challenges in terms of environmental adaptability, such as waves, temperature, humidity, and high salt corrosion. As shown in Table 1, the proposed solutions for the key influencing factors of environmental adaptability were listed. The implementation and feasibility verification of the solutions will be elaborated in detail in the second manuscript “Circular Array Fiber-optic Sub-sensor for Large-area Bubble Observation, Part II: Array Assemble and Field Experimental Validation”.

## Figures and Tables

**Table 1:** Key improvement schemes for environmental adaptability of the prototype of the integrated BBS.

Improvement Project	Improvement Plan Conception
Platform stability cause by waves	Add attitude sensors and implement motion compensation before mapping the omnidirectional bubble image and the panoramic bubble image.
Adaptability to temperature and humidity	The transparent support column is embedded with resistance wire, equipped with a temperature sensor and control circuit, and a desiccant is provided.
Adaptability to high salt corrosion	Make the acrylic glass (polymethyl methacrylate, PMMA) in the prototype more weather-resistant acrylic glass.

**Figure A1:**
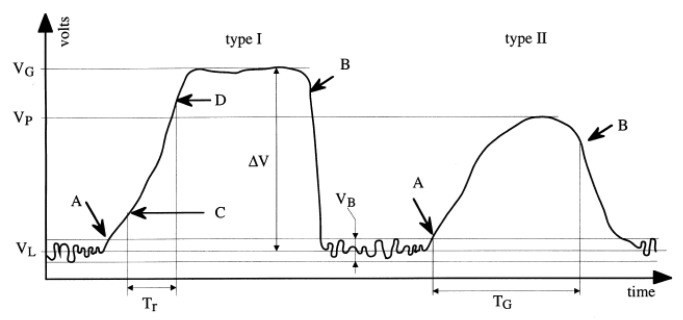
Sketch of characteristic events detected on bubble signatures.

**Figure A2:**
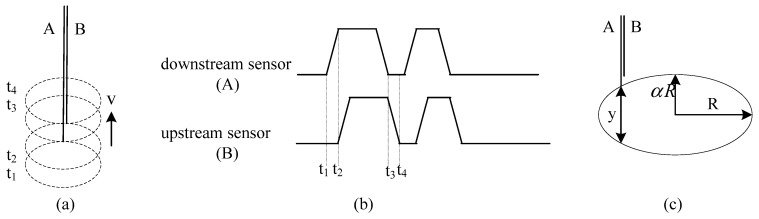
Illustration of signals obtained by the dual-array element: (**a**) the principle of piercing bubble of dual-array element; (**b**) the signal of dual-array element; (**c**) the chord of piercing bubble.

**Figure A3:**
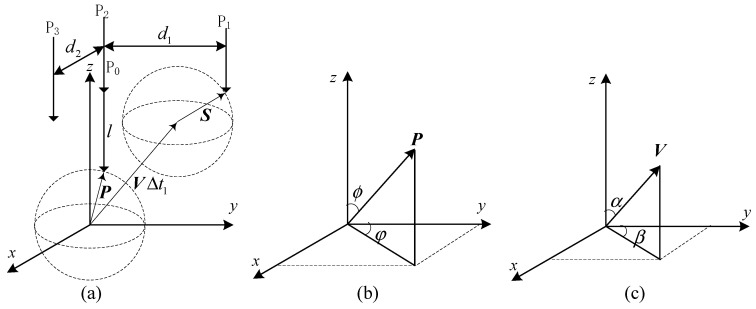
The mathematic model of four-array element. (**a**) the model of piercing bubble of four-array element; (**b**) the position of four-array element; (**c**) the position of bubble velocity.

**Figure A4:**
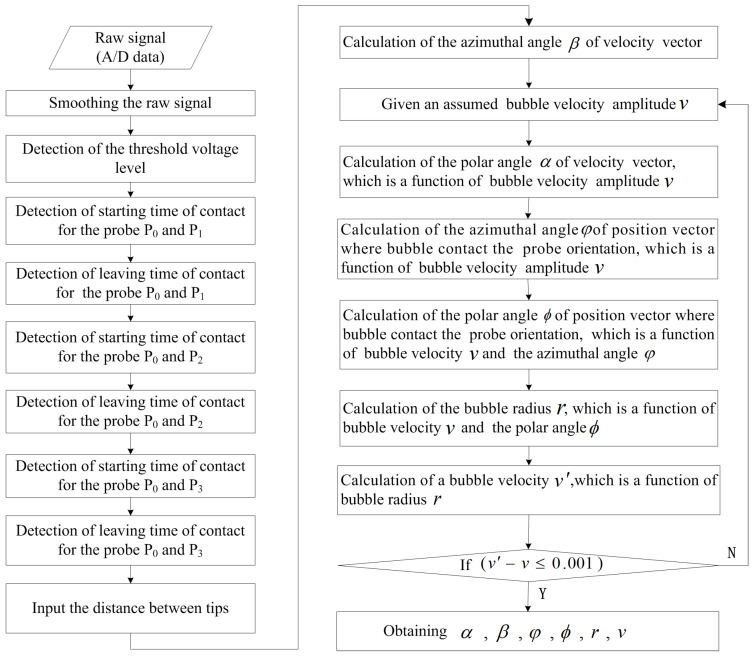
Flow chart of the signal processing employed.

**Figure A5:**
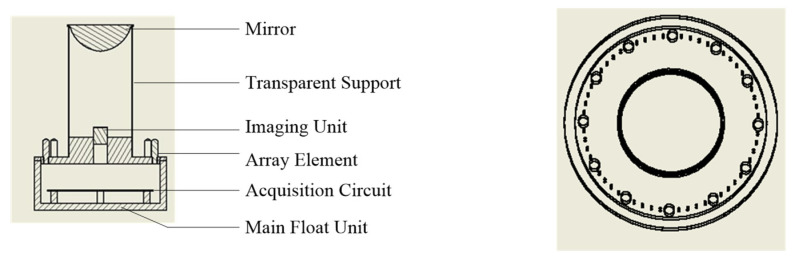
Structural schematic of BBS. (**a**) Cross-sectional view; (**b**) Top view.

**Figure A6:**
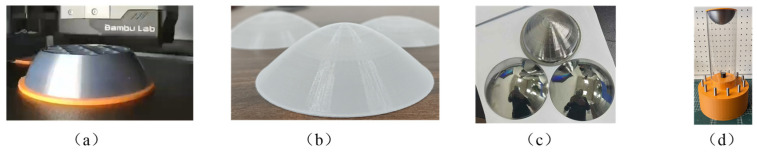
Prototype of BBS: (**a**) snapshot of 3D printing for reflector; (**b**) reflector after3D printing; (**c**) reflector after electroplating; (**d**) assembled BBS prototype.

**Figure A7:**
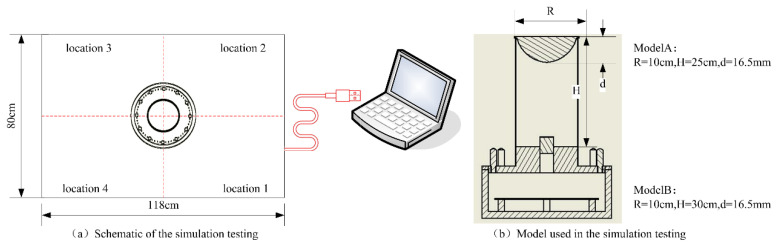
Schematic of the simulation testing and experimental equipment.

**Figure A8:**
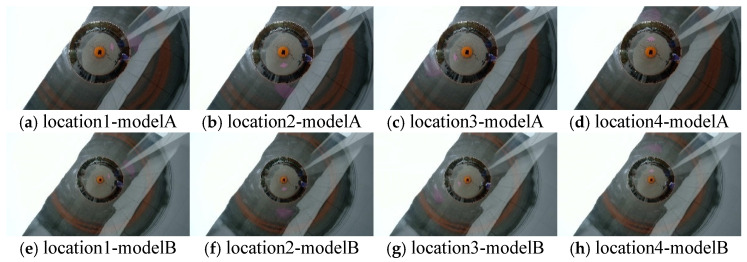
Original images corresponding to two models of BBS.

## Data Availability

The original contributions presented in this study are included in the article. Further inquiries can be directed to the corresponding author.

## Appendix A

### Appendix A.1. Algorithm for Calculating Bubble Parameters from Voltage Signals Acquired by Single-Array Element (Applicable to This Manuscript)

The bubble chord s is given by

(A1) $$s=V\times T_{d}$$

where *V* represents the velocity of the bubble relative to the probe, *T_d_* denotes the gas dwell time of the bubble at the probe, and *s* indicates the chord length of the bubble.

Regarding how to accurately obtain the velocity of the bubble, Rojas and Loewen [23] improved the calibration experimental apparatus based on previous research, making it applicable not only to unidirectional gas–liquid two-phase flow observation systems but also to bubble plume observation systems with unknown bubble paths. Through the analysis and fitting of extensive data, they found that the bubble velocity conforms to the power-law equation shown in Equation (A2) or the logarithmic form presented in Equation (A3):

(A2) $$V=10^{b}T_{r}^{m}$$

(A3) $$\log(V)=m\times \log(T_{r})+b$$

where *V* represents the velocity of the bubble relative to the probe, *Tr* denotes the rise time of the bubble, and b and m are constants determined based on calibration data, which can be calculated using the least squares method.

Therefore, to calculate the bubble chord length, in addition to the calibration coefficients *m* and *b* for each probe, it is also necessary to determine two key parameters: the gas dwell time *T_d_* and the bubble rise time *Tr.*

As shown in Figure A1, the identification of the gas dwell time *T_d_* is based on the determination of the dates of probe entry T_A_ and exit T_B_ through each bubble. As referred to in [24], and discarding probe geometries delivering pre-signals, the very beginning of the signal rise corresponds to the detection of the disturbed interface location. Hence, to identify the bubble signatures, only two quantities have to be evaluated: the amplitude corresponding to a fully wetted probe V_L_ and the peak to peak noise amplitude V_B_. Since discrimination is not possible within the noise, the presence of a bubble is detected whenever the signal exceeds the level V_T_ = V_L_ + CS1 V_B_, where CS1 is a security coefficient accounting for the randomness of the noise. For a Gaussian noise, the coefficient CS1, which varies between 0.5 and 1, is typically set to 0.75, so that the probability of interpreting a noise peak as a bubble is less than 0.005. Note also that since bubble signatures lower than level V_T_ are ignored, it is recommended to ensure a good enough signal-to-noise ratio (SNR) to minimize V_T_. The actual entry date T_A_ is determined by the occurrence of a signal anterior to the beginning of the event detected, and whose amplitude exceeds the base level V_L_ by an amount of V_B_/2. Hence, T_A_ is determined by the parameters V_L_ and V_B_; its dependency on CS1 being only conditional, the above procedure differs from absolute thresholding. For the exit date T_B_, an analysis of the shape of the signature is first required. If a plateau exists, the de-wetting process has been almost completed (neglecting here the influence of tiny droplets stuck on the fiber), and the ensuing wetting process produces a steep signal decrease as shown by the type I signature in Figure A1. Denoting V_G_ as the mean plateau level, the exit date T_B_ corresponds to an amplitude VG-CS3 (VG-VL), where according to the water-entry responses observed in well-controlled conditions (as referred to in [24]), CS3 ranges from a few percent up to 30%. The situation is less clear for low amplitude, usually bell-shaped, signatures, such as the type II signal in Figure A2, which is characteristic of incomplete probe drying. It has been decided to apply again the above criteria, replacing V_G_ with the peak amplitude V_P_. To finely adjust the parameter CS3 in this case, more work is needed to locate the rear interface on type II signatures. At present, values in the range of 5 ± 20% for CS3 are currently used, the default value being 10%. In all cases, with V_G_ or V_P_ being computed for each event, CS3 is the only free (but strongly bounded) parameter intervening in the detection of T_B_.

The waveform analysis is based on a sub-sampling controlled by a single amplitude parameter WA. Starting from the event T_A_, sample points are tagged whenever the amplitude difference with the previous tagged point is at least WA. This sub-set of tagged events reproduces the shape of the waveform with a low number of data. A compromise must be sought between a refined-enough tagging leading to acceptable uncertainties on VG or VP and a reduced number of computations to ensure real-time detection by the processor. As referred to in [25], to estimate this optimum, it is necessary to perform the tests on actual signals collected in bubble flows and combined with random Gaussian noise. In this test, ΔV is defined as the maximum amplitude deference observed for a wet and a dry probe, and the WA/ΔV will meet the constraints under which the relative uncertainty on the gas dwell time $T_{G}=T_{B}-T_{A}$ is minimized.

### Appendix A.2. Algorithm for Calculating Bubble Parameters from Voltage Signals Acquired by Dual-Array Element

As referred to in [26], there are two sensors in dual-array elements: the downstream sensor, seen as A in Figure A2a, and the upstream sensor, seen as B in Figure A2a, which are aligned in the same direction by small distance. Each sensor is constructed with two optical fibers: one fiber for transmitting light and another for receiving it. If the sensor tip is surrounded by liquid, there is very little light reflected back, but if it pierces a gas bubble, the light is almost totally reflected. The reflected light is transmitted back to the signal-conditioning module via the second optical fiber. The signal measured is the amount of light detected at the signal conditioning module; thus, a low signal level indicates water, whereas a high level indicates air.

The typical output signals from the dual-array element are shown in Figure A2b. With the acquired signals from the sensor, the bubble velocity *v* is given by

(A4) $$v=\frac{1}{2}\left(\frac{s}{t_{2}-t_{1}}+\frac{s}{t_{4}-t_{3}}\right)$$

where s is the distance between the downstream sensor and the upstream sensor, $t_{1}$ is the time when the bubble impacts the downstream sensor, $t_{2}$ is the time when the bubble impacts the upstream sensor, $t_{3}$ is the time the downstream sensor takes to fully penetrate the bubble, and $t_{4}$ is the time the upstream sensor takes to fully penetrate the bubble.

The void fraction can be also measured by the dual-array element, $\alpha_{P}$ is given by

(A5) $$\alpha_{P}=\frac{1}{T}\sum_{i=1}^{N_{B}}{\left(T_{g}\right)}_{i}$$

where ${\left(T_{g}\right)}_{i}$ is the gas residence time of the *i*th bubble, $N_{B}$ is the total number of bubbles for which the gas residence time was measured, and T is the total sampling time.

Furthermore, the probability density function of the diameters of bubbles can be found from the probability density function of the chord of bubbles:

(A6) $$P_{C}\left(y\right)=\int_{\frac{y}{2\alpha }}^{\infty }\frac{y}{\alpha^{2}R^{2}}P_{p}\left(R\right)dR$$

where $P_{p}\left(R\right)$ is the probability density function of the sizes of bubbles, R is the radius of bubbles, and α is the ratio of semi-major axis and semi-minor axis of the ellipse, as shown in Figure A2c.

### Appendix A.3. Algorithm for Calculating Bubble Parameters from Voltage Signals Acquired by Four-Array Element

The algorithm for the four-array element relies on the probe configuration. A typical mathematic model is shown in Figure A3. There are four sensors including the leading tip P0 and three lateral tips, P1–P3. P0 and P2 are aligned along the z axis with a distance of l, and P1 and P3 are at distances $d_{1}$ and $d_{2}$ from P0 along the x and y axis, respectively. Each pair of the front sensor and one rear sensor can be considered as a dual-array element, which means there are three four-array elements in a dual-array element, and the principle of the four-array element is similar to the dual-array element.

The algorithms for the four-array element are shown in Figure A4. The signal processing is composed of four processes as described below: (1) the smoothing process; (2) the determination of thresholds process; (3) the detection of time intervals of bubble impact on the tips; (4) the calculation of velocity vector, the pierced position vector, and other required parameters. Since the first two processes involve the true signal collected by probes, this section will give the key points of the last two processes.

## Appendix B

### Appendix B.1. Sensor Prototype Fabrication

The fabrication of the BBS sensor primarily involves the structural formation of the optical system for the panoramic bubble imaging sub-sensor, the fiber-optic array sub-sensor elements, and the sensor packaging. Since the sensor packaging structure is designed for the integration of two sub-sensors, it follows the structural diagram shown in Figure A5a. A one-piece design for three units is adopted, with the primary float body modeled after a miniature buoy. The main structural components of the panoramic bubble imaging sub-sensor optical system include the reflection mirror, transparent support, and imaging unit. The mirror reflects the surrounding bubbles’ object points and forms an image on the corresponding detector plane of the imaging unit. The transparent support columns provide structural support for the mirror and imaging unit, while the primary float body provides a watertight space for the acquisition circuitry and serves as a platform for the two sub-sensors. The array elements, as shown in the schematic of Figure A6b, are evenly distributed around the primary float body at a 30° angle. The initial prototypes of the sub-sensors and units are shown in Figure A6.

### Appendix B.2. Sensor Prototype Testing

To validate the feasibility of the sensor’s ability to cover a large area in a single data acquisition, a simulated experimental scenario as shown in Figure A7 was constructed. This scenario consists mainly of a panoramic bubble imaging sub-sensor model and a PC. The panoramic bubble imaging sub-sensor transmits the acquired bubble ring images to the PC for data collection via a USB cable. To minimize reflections from the ground and simulate the characteristics of both the measurement background and the target object, the detection area includes both high-similarity background and target objects, as well as objects with distinct textural features. A rectangular rough ground environment measuring 118 cm × 80 cm was chosen as the test region for this purpose. Considering the varying target-line clarity at the four corner positions of the detection area, red test boards were placed at these four corners to improve the accuracy of subsequent image calibration, circular panoramic bubble image mapping, and other image recognition processes. These four test points were labeled as location 1 to location 4. Moreover, to account for errors introduced during the fabrication and assembly process, two different sensor models with varying reflector installation heights were tested, as illustrated in Figure A7b.

Figure A8 shows the original images captured by the sensor at locations 1~4. Analysis of the images captured by the two models leads to the following conclusions: (1) Since the sensor captures images reflected from transparent support columns and mirrors, the acquired images are mirrored along the horizontal centerline of the target area. This mirroring effect should be taken into account when calculating the sensor’s coverage area in later stages. (2) The acquired images are panoramic ring-shaped images. Both models are capable of capturing images outside the test region, indicating that the sensor has a large-area sampling capability.
